# The Septins Function in G1 Pathways that Influence the Pattern of Cell Growth in Budding Yeast

**DOI:** 10.1371/journal.pone.0002022

**Published:** 2008-04-23

**Authors:** Thea A. Egelhofer, Judit Villén, Derek McCusker, Steven P. Gygi, Douglas R. Kellogg

**Affiliations:** 1 Department of Molecular, Cell and Developmental Biology, University of California Santa Cruz, Santa Cruz, California, United States of America; 2 Department of Cell Biology, Taplin Biological Mass Spectrometry Facility, Harvard Medical School, Boston, Massachusetts, United States of America; University of Edinburgh, United Kingdom

## Abstract

The septins are a conserved family of proteins that have been proposed to carry out diverse functions. In budding yeast, the septins become localized to the site of bud emergence in G1 but have not been thought to carry out important functions at this stage of the cell cycle. We show here that the septins function in redundant mechanisms that are required for formation of the bud neck and for the normal pattern of cell growth early in the cell cycle. The Shs1 septin shows strong genetic interactions with G1 cyclins and is directly phosphorylated by G1 cyclin-dependent kinases, consistent with a role in early cell cycle events. However, Shs1 phosphorylation site mutants do not show genetic interactions with the G1 cyclins or obvious defects early in the cell cycle. Rather, they cause an increased cell size and aberrant cell morphology that are dependent upon inhibitory phosphorylation of Cdk1 at the G2/M transition. Shs1 phosphorylation mutants also show defects in interaction with the Gin4 kinase, which associates with the septins during G2/M and plays a role in regulating inhibitory phosphorylation of Cdk1. Phosphorylation of Shs1 by G1 cyclin-dependent kinases plays a role in events that influence Cdk1 inhibitory phosphorylation.

## Introduction

G1 cyclins bind and activate cyclin-dependent kinases (CDKs) to initiate cell cycle events in G1 [1], [2]. In budding yeast, an important function of the G1 cyclins is to initiate growth of a new daughter cell [3]–[6]. There are two CDKs that function during G1 in budding yeast, called Cdk1 and Pho85, which are activated by numerous different cyclins [1]. Cdk1 is activated by Cln1, Cln2, and Cln3, while Pho85 is activated by Pcl1 and Pcl2, as well as a number of additional cyclins that do not appear to directly regulate G1 events. The G1 cyclins are highly redundant. For example, loss of Cln3 or Cln1 and Cln2 causes delayed initiation of bud growth, but loss of all three cyclins leads to a failure to initiate bud growth [4], [5]. Similarly, loss of Pcl1 and Pcl2 causes no obvious phenotype, but cells lacking Pcl1, Pcl2, Cln1, and Cln2 fail to form a focused bud neck and show severe defects in the pattern of bud growth [3], [7], [8]. The extensive redundancy of G1 cyclins implies the existence of multiple overlapping pathways that control G1 events.

The mechanisms used by the G1 cyclins to control G1 events are poorly understood. *CLN1* and *CLN2* interact genetically with a member of the septin family of proteins, which suggests that septins may function in events controlled by G1 cyclins [9]. There are five septins expressed in vegetatively growing yeast cells that are referred to as Cdc3, Cdc10, Cdc11, Cdc12, and Shs1 [10]–[12]. The septins form a tight complex with each other and are localized to the bud neck [13]–[16]. Loss of Cdc3, Cdc10, Cdc11, or Cdc12 causes cells to arrest or delay at G2/M while polar bud growth continues, which results in aberrant growth of highly elongated cells [11]. In contrast, loss of Shs1 causes only a mild and weakly penetrant elongated bud phenotype, and the role of this septin has therefore been unclear [17], [18]. The septins have been proposed to function as cytoskeletal elements, scaffolds, factors involved in vesicle targeting or fusion, or as a diffusion barrier that prevents movement of membrane proteins between the mother and daughter cell [10], [12], [19]–[30]. Septins have domains that bind to GTP and phosphoinositides, but the functions of these domains are poorly understood [10].

Although loss of septin function causes a G2/M arrest in budding yeast, there is some evidence that the septins may also carry out functions early in the cell cycle. A unique allele of Cdc12 is synthetically lethal with *cln1Δ cln2Δ,* and the septins become localized to the site of bud emergence in G1 [9], [16]. In addition, loss of septin function causes the newly emerging bud to be slightly elongated and to have a misshapen bud neck [31], [32]. Important functions for the septins during G1 may be masked by the existence of redundant pathways that control G1 events.

In this study, we tested whether septins function in pathways controlled by G1 cyclins and identified a septin-dependent pathway that controls the pattern of cell growth early in the cell cycle. Moreover, we found that the Shs1 septin shows strong genetic interactions with G1 cyclins and is a direct target of CDKs associated with G1 cyclins. However, phosphorylation of Shs1 does not appear to play a role in septin-dependent pathways that control the pattern of cell growth during G1. Rather, Shs1 phosphorylation appears to influence events that regulate inhibitory phosphorylation of Cdk1 at the G2/M transition. Phosphorylation of Shs1 by G1 cyclin-dependent kinases could therefore play a role in mechanisms that help link entry into mitosis to successful completion of G1 events.

## Results

### Genetic interactions suggest that the Shs1 septin may function in G1 pathways

**Figure 1:**
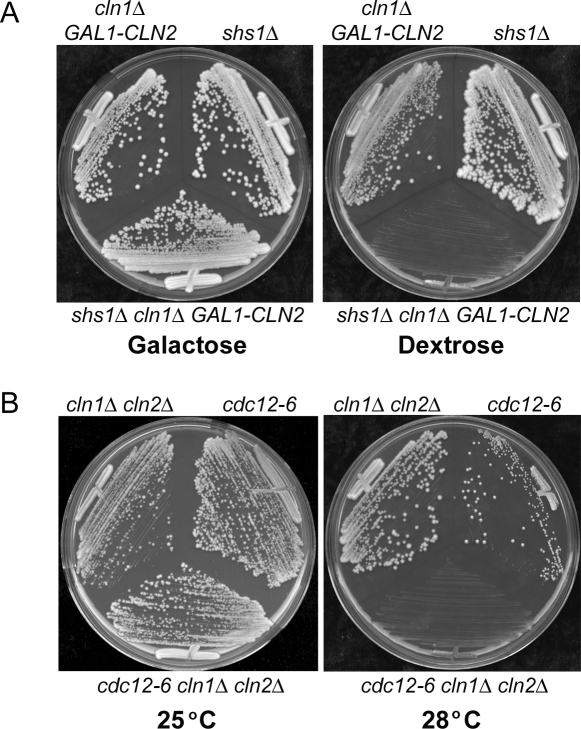
*shs1Δ* and *cdc12-6* are synthetically lethal with *cln1Δ cln2Δ.* (A) Growth of *cln1Δ GAL1-CLN2*, *shs1Δ,* and *shs1Δ cln1Δ GAL1-CLN2* cells was monitored on YP media containing galactose or dextrose at 30°C. (B) Growth of *cln1Δ cln2Δ*, *cdc12-6*, and *cdc12-6 cln1Δ cln2Δ* cells was monitored on YPD plates at 25°C and 28°C.

Since an allele of *CDC12* was previously found to show synthetic lethal interactions with *cln1Δ cln2Δ*, we tested whether this is also true for *shs1Δ* [9]. We were unable to recover viable *cln1Δ cln2Δ shs1Δ* haploids, which suggested that *shs1Δ* is synthetically lethal with *cln1Δ cln2Δ*. To further confirm that *shs1Δ* is synthetically lethal with *cln1Δ cln2Δ*, we generated a *shs1Δ cln1Δ GAL1-CLN2* strain, in which the expression of *CLN2* could be repressed by dextrose. This strain grew normally on galactose, but failed to grow on dextrose (Figure 1A). Loss of *SHS1* was not synthetically lethal with *pcl1Δ pcl2Δ*. To test for additional genetic interactions between the septins and *cln1Δ cln2Δ*, we determined whether a commonly used temperature sensitive allele of *CDC12*, *cdc12-6*, showed a genetic interaction with *cln1Δ cln2Δ*. In this case, we found that *cln1Δ cln2Δ* lowered the restrictive temperature of the *cdc12-6* allele (Figure 1B). Thus, several septins and septin alleles show strong genetic interactions with G1 cyclins. The finding that *SHS1* is essential for viability in *cln1Δ cln2Δ* cells but not in wild type cells suggests that it may function in redundant pathways that control G1 events.

### Septins are required for the normal pattern of cell growth in *cln1Δ cln2Δ* cells

**Figure 2:**
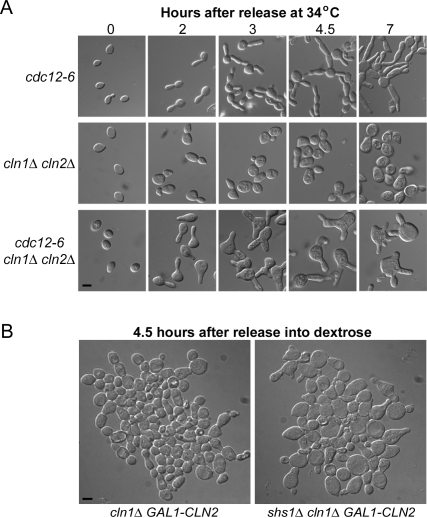
Loss of Cdc12 or Shs1 in *cln1Δ cln2Δ* cells causes defects in formation of the bud neck and the pattern of growth. (A) *cdc12-6*, *cln1Δ cln2Δ*, and *cdc12-6 cln1Δ cln2Δ* cells were synchronized by centrifugal elutriation and released into YPD media at 34°C, the restrictive temperature for the *cdc12-6* allele. Micrographs were taken at the indicated timepoints after release. Bar, 5 µm for all panels (B) Cells of the indicated genotypes were grown to log phase in YP media containing galactose and transferred to YPD media at 30°C. Micrographs were taken 4.5 hours after release into YPD media. Bar, 5 µm for both panels.

We used the *cdc12-6 cln1Δ cln2Δ* cells and the *shs1Δ cln1Δ GAL1-CLN2* cells to determine the consequences of a loss of septin function in cells that lack Cln1 and Cln2. We first used centrifugal elutriation to synchronize *cdc12-6*, *cln1Δ cln2Δ*, and *cdc12-6 cln1Δ cln2Δ* cells in early G1 and then released them at the restrictive temperature for the *cdc12-6* allele (34°C). The *cdc12-6 cln1Δ cln2Δ* cells were unable to form buds with normal morphology and largely failed to direct growth to the daughter bud (Figure 2A). The cells were also unable to form a normal bud neck with a well-defined constriction between the mother and daughter cell.

We next shifted rapidly growing *shs1Δ cln1Δ GAL1-CLN2* cells from galactose to dextrose. After 4.5 hours, some cells arrested as large unbudded cells, while others arrested with small poorly formed buds that lacked a well-defined bud neck (Figure 2B). We were unable to synchronize the *shs1Δ cln1Δ GAL1-CLN2* cells by centrifugal elutriation because cells carrying *shs1Δ* form clumps. These results demonstrate that loss of Shs1 or Cdc12 in cells that lack Cln1 and Cln2 causes defects in the pattern of cell growth that are distinct from the defects caused in wild type cells.

### The Shs1 septin is required for septin localization in cells that lack Cln1 and Cln2

**Figure 3:**
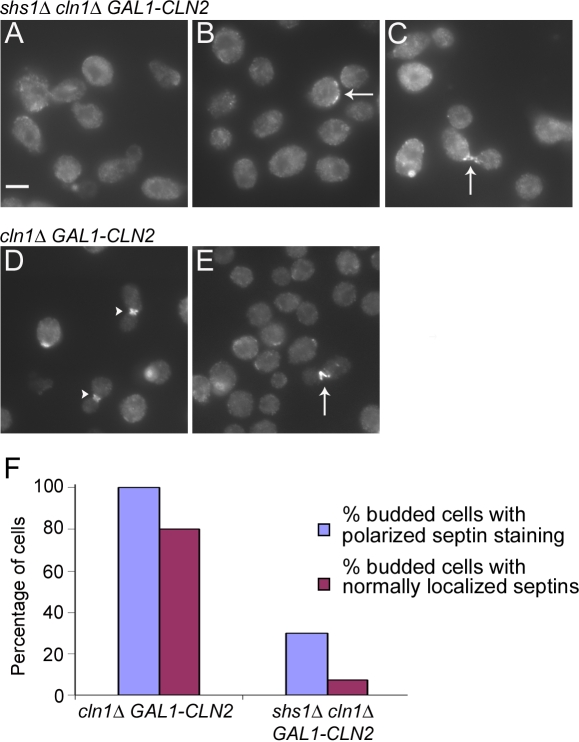
Shs1 is required for normal localization of the Cdc11 septin in *cln1Δ cln2Δ* cells. (A–C) *shs1Δ cln1Δ GAL1-CLN2* cells and (D–E) *cln1Δ GAL1-CLN2* control cells were grown to log phase in YP media containing galactose and switched to YPD media for 4.5 hours. Cdc11 localization was determined with an anti-Cdc11 antibody. Arrows point to abnormal Cdc11 localization. The arrow head points to normal Cdc11 localization. Bar, 5 µm for all panels. (F) The percentage of budded cells in *cln1Δ GAL1-CLN2* and *shs1Δ cln1Δ GAL1-CLN2* cells that had any polarized Cdc11 localization in the mother or daughter cell was determined, including cells that had polarized but abnormal localization of Cdc11. The percentage of budded cells that had normal Cdc11 localization at the bud neck was also determined. 200 cells were counted for each strain.

Loss of Cdc3, Cdc10, Cdc11 or Cdc12 results in rapid loss of localization of the septins; however, loss of Shs1 has no effect on septin localization [16], [33], [34]. Since Shs1 is essential for viability in *cln1Δ cln2Δ* cells, we tested whether Shs1 is essential for localization of the other septins in cells that lack Cln1 and Cln2. We shifted *shs1Δ cln1Δ GAL1-CLN2* and *cln1Δ GAL1-CLN2* control cells into dextrose for 4.5 hours and used immunofluorescence to test for Cdc11 localization. Most *shs1Δ cln1Δ GAL1-CLN2* cells showed a complete failure to localize Cdc11 (Figure 3A), although some unbudded cells had diffuse Cdc11 localization over one end of the cell (arrow, Figure 3B). Of the few budded *shs1Δ cln1Δ GAL1-CLN2* cells, only 30% had polarized Cdc11 localization in the mother or daughter cell and the Cdc11 localization in the majority of these cells was aberrant (arrow, Figure 3C). Only 7.5% of budded cells had normal Cdc11 localization (Figure 3F). In *cln1Δ GAL-CLN2* control cells, 100% of budded cells had polarized Cdc11 localization and 80% had normal Cdc11 localization (arrowhead, Figure 3D and 3F). In addition, the defects in Cdc11 localization that were observed in cells that lack Cln1 and Cln2 were less severe than the defects observed in cells that lack Shs1, Cln1, and Cln2 (arrow, Figure 3E). These results demonstrate that Shs1 is required for the normal localization of Cdc11 in cells that lack Cln1 and Cln2.

### Shs1 is a target of multiple CDK-cyclin complexes during G1

**Figure 4:**
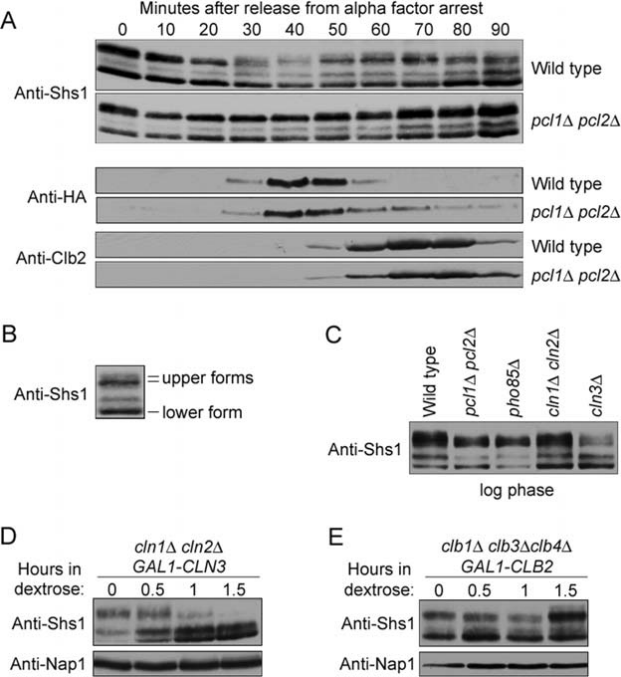
Shs1 phosphorylation is dependent upon multiple CDK-cyclin complexes. (A) Wild type and *pcl1Δ pcl2Δ* cells were arrested in G1 by the addition of α factor. The cells were released from the arrest and time points were taken every 10 minutes. The behavior of Shs1, Cln2-3XHA, and Clb2 was followed by Western blotting. The Cln2-3XHA and Clb2 time courses are from a different experiment, but the timing of Shs1 phosphorylation during the cell cycle was similar in both experiments. (B) The sample taken from wild type cells at 60 minutes in the time course shown in Figure 4A was labeled to indicate the different isoforms of Shs1. (C) Western blot analysis of Shs1 phosphorylation in log phase populations of wild type, *pcl1Δ pcl2Δ*, *pho85Δ*, *cln1Δ cln2Δ*, and *cln3Δ* cells. A loading control is not shown because slightly different amounts of protein were loaded to obtain exposures that allow accurate comparison of the relative amounts of phosphorylation isoforms. (D) *cln1Δ cln2Δ GAL1-CLN3* and (E) *clb1Δ clb3Δ clb4Δ GAL1-CLB2* cells were grown to log phase in YP media containing galactose and released into YPD media for the indicated times. The behavior of Shs1 was followed by Western blotting. The same samples were probed with an anti-Nap1 antibody to provide loading controls.

The finding that Shs1 is essential for viability and for septin localization in cells that lack Cln1 and Cln2 suggested that it may function in pathways regulated by G1 cyclins. To test this idea, we first assayed the phosphorylation state of Shs1 during the cell cycle in wild type and *pcl1Δ pcl2Δ* cells. Previous work demonstrated that Shs1 undergoes phosphorylation that can be detected by a shift in electrophoretic mobility [13]. Wild type and *pcl1Δ pcl2Δ* cells were released from a G1 arrest and phosphorylation of Shs1 was monitored during the cell cycle by Western blotting (Figure 4A). Levels of the G1 cyclin Cln2 and the mitotic cyclin Clb2 were also monitored to provide markers for cell cycle progression. In wild type cells, multiple phosphorylated forms of Shs1 could be detected during the cell cycle. We refer to the most slowly migrating forms of Shs1 as the upper forms, and the most rapidly migrating form of Shs1 as the lower form (Figure 4B). At 30 minutes, the upper form of Shs1 underwent further hyperphosphorylation that could be detected as a shift in electrophoretic mobility and a broadening of the electrophoretic band. The hyperphosphorylation occurred concurrently with synthesis of the G1 cyclin Cln2 and prior to synthesis of Clb2, which indicated that it was initiated during G1/S phase. Similar hyperphosphorylated forms of Shs1 failed to appear when Cln2 was synthesized in *pcl1Δ pcl2Δ* cells (Figure 4A).

We next tested whether other G1 cyclin/CDK complexes also play a role in phosphorylation of Shs1. To do this, we first assayed Shs1 phosphorylation in log phase populations of *pcl1Δ pcl2Δ*, *pho85Δ*, *cln1Δ cln2Δ*, and *cln3Δ* cells (Figure 4C). Consistent with the results from synchronized cells, the upper form of Shs1 failed to become fully hyperphosphorylated in rapidly growing *pcl1Δ pcl2Δ* cells. Similarly, the upper form of Shs1 failed to become fully hyperphosphorylated in *pho85Δ* cells. In *cln1Δ cln2Δ* cells, there was little effect on the hyperphosphorylated upper forms of Shs1, but we consistently observed an increase in the hypophosphorylated lower form of Shs1. In *cln3Δ* cells, the hyperphosphorylated upper forms of Shs1 were significantly reduced. We also assayed Shs1 phosphorylation in *cln1Δ cln2Δ GAL1-CLN3* cells after repression of *CLN3* expression with dextrose. The hyperphosphorylated upper forms of Shs1 were rapidly and significantly reduced when the *cln1Δ cln2Δ GAL1-CLN3* cells were transferred to dextrose (Figure 4D). Previous work has shown that Cln3 plays an important role in pathways that help trigger transcription of Pcl1, Pcl2, Cln1, and Cln2, which may explain the strong effects of loss of Cln3 on Shs1 phosphorylation [3], [35], [36].

To test whether Shs1 hyperphosphorylation is primarily dependent upon G1 cyclins, we also assayed the effects of depletion of mitotic cyclins on Shs1 phosphorylation. As with G1 cyclins, there are multiple redundant mitotic cyclins, which are called Clb1, Clb2, Clb3, and Clb4 [37]. Clb2 is the most important mitotic cyclin and cells can be made dependent upon Clb2 for viability by deleting the genes for the other mitotic cyclins. We assayed Shs1 phosphorylation in *clb1Δ clb3Δ clb4Δ GAL1-CLB2* cells after a shift to dextrose to repress Clb2 transcription (Figure 4E). The upper phosphorylated forms of Shs1 were not significantly reduced when cells were depleted of all mitotic cyclins. Depletion of mitotic cyclins caused Shs1 to accumulate in hyperphosphorylated forms because mitotic cyclins are required for repression of G1 cyclins, and cells therefore arrest with high levels of G1 CDK activity (Figure 4E) [38]. Together, these results demonstrate that Cdk1 and Pho85 associated with G1 cyclins are required for full hyperphosphorylation of Shs1 in vivo.

### Pho85-Pcl1 and Cdk1-Cln2 can directly hyperphosphorylate Shs1

**Figure 5:**
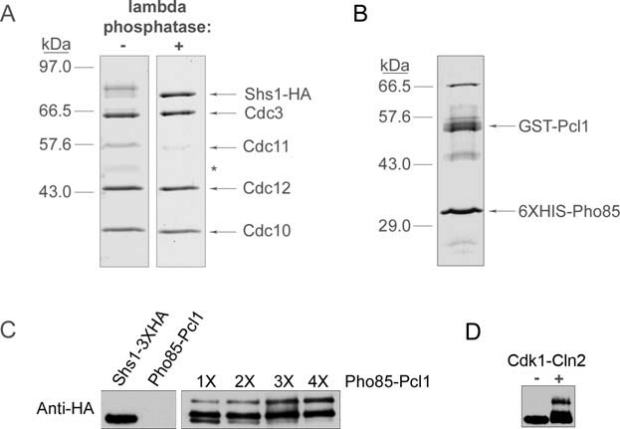
Pho85-Pcl1 can hyperphosphorylate Shs1 in vitro. (A) A Coomassie blue-stained polyacrylamide gel that shows purified Shs1-3XHA with or without treatment with lambda phosphatase. The asterisk marks a background band that is present in some septin purifications. (B) A Coomassie blue-stained polyacrylamide gel that shows purified 6HIS-Pho85/GST-Pcl1 co-purified from bacteria. A number of background bands co-purify with 6HIS-Pho85/GST-Pcl1. The band migrating around 65 KD is most likely a heat shock protein. (C) Purified dephosphorylated Shs1-3XHA complexes were incubated with increasing amounts of purified 6XHIS-Pho85/GST-Pcl1 in the presence of ATP for 1 hour at 30°C (right panel). The reactions were then loaded onto a 10% SDS-polyacrylamide gel and the phosphorylation state of Shs1 was monitored by Western blotting. As a control, purified Shs1-3XHA and purified 6XHIS-Pho85/GST-Pcl1 were incubated individually with ATP (left panel). (D) Purified dephosphorylated Shs1-3XHA complexes were incubated with purified 3XHA-Cln2/Cdk1 complexes for 30 minutes at 30°C. The reactions were then loaded onto a 10% SDS-polyacrylamide gel and the phosphorylation state of Shs1 was monitored by Western blotting.

We next addressed whether G1 CDKs can directly hyperphosphorylate Shs1. We initially focused on phosphorylation of Shs1 by Pho85 because active Pho85-Pcl1 complexes can be readily purified after expression in bacteria [39]. In addition, Shs1 was previously identified in a high-throughput screen for proteins that are phosphorylated by Pho85-Pcl1 or Pho85-Pho80 [39]. In this screen, Shs1 was found to be a highly specific substrate of Pho85-Pcl1 when compared with Pho85-Pho80; however, the experiments did not determine whether Pho85-Pcl1 is capable of generating the fully hyperphosphorylated upper forms of Shs1 that are observed in vivo. To carry out a more detailed analysis of Shs1 phosphorylation, we used immunoaffinity chromatography to purify Shs1-3XHA from yeast cells to use as a substrate for kinase reactions. The immunoaffinity purification was carried out in the presence of 1M KCl to remove all but the most tightly associated proteins. Under these conditions, Shs1-3XHA was found in a complex with the other septins, as previously reported (Figure 5A, first lane) [13]. We also treated Shs1-3XHA with lambda phosphatase during the purification to generate the fully dephosphorylated form of Shs1 (Figure 5A, second lane). 6XHIS-Pho85 and GST-Pcl1 were co-expressed in bacteria and purified by affinity chromatography (Figure 5B). Incubation of purified Shs1-3XHA with purified 6XHIS-Pho85/GST-Pcl1 caused Shs1-3XHA to shift to two discrete hyperphosphorylated forms (Figure 5C). The fully hyperphosphorylated form of Shs1 generated in vitro was similar to the hyperphosphorylated upper form of Shs1 observed in vivo. Since Cln cyclins are also required in vivo for full Shs1 phosphorylation, we tested whether Cdk1-Cln2 can hyperphosphorylate Shs1. We used immunoaffinity chromatography to purify Cdk1/3XHA-Cln2 from yeast. Incubation of Cdk1/3XHA-Cln2 with the purified septin complexes caused Shs1 to undergo a shift in electrophoretic mobility similar to the shift induced by Pho85-Pcl1. These results show that Pho85-Pcl1 and Cdk1-Cln2 are capable of generating at least some of the hyperphosphorylated forms of Shs1 observed in vivo.

### Pho85-Pcl1 phosphorylates Shs1 on consensus and non-consensus sites

**Figure 6:**
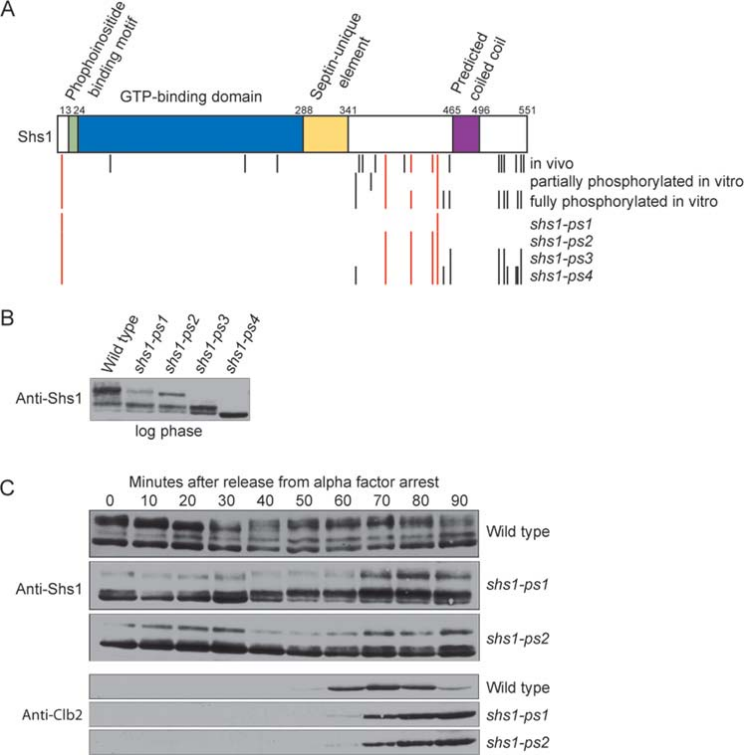
Mapping and analysis of Pho85-Pcl1 phosphorylation sites on Shs1. (A) The distribution of in vitro and in vivo phosphorylation sites on Shs1, and a summary of the phosphorylation sites mutated in *shs1-ps* mutants. Minimal CDK-consensus sites (SP or TP) are denoted in red. (B) Shs1 phosphorylation was analyzed in log phase populations of wild type, *shs1-ps1*, *shs1-ps2*, *shs1-ps3*, and *shs1-ps4* cells by Western blotting. A loading control is not shown because slightly different amounts of protein were loaded to obtain exposures that allow accurate comparison of the relative amounts of phosphorylation isoforms. (C) Wild type, *shs1-ps1*, and *shs1-ps2* cells were released from an α factor arrest and samples were taken every 10 minutes. Shs1 phosphorylation and Clb2 levels were monitored by Western blotting.

To further confirm that Pho85-Pcl1 phosphorylates Shs1 in vivo, we used mass spectrometry to map phosphorylation sites on both of the phosphorylated forms of Shs1 generated in vitro. We also mapped in vivo phosphorylation sites and then compared these to the in vitro sites. To map in vivo phosphorylation sites, we purified Shs1-3XHA from yeast cells by immunoaffinity chromatography in the presence of high salt and high concentrations of phosphatase inhibitors. Excellent sequence coverage was obtained for all of the mapping experiments (greater than 85%). The results are summarized in Table 1 and Figure 6A. Five phosphorylation sites were identified on the partially hyperphosphorylated form of Shs1 generated in vitro, and twelve were identified on the fully hyperphosphorylated form. Of the five minimal CDK consensus sites in Shs1 (SP or TP), three were found to be phosphorylated in the partially hyperphosphorylated form of Shs1 and four were found in the fully hyperphosphorylated form. In each case where a CDK consensus site was not detected on Shs1 phosphorylated in vitro, the peptides containing that site were not covered by the mapping so it is possible that the site was phosphorylated. The fully hyperphosphorylated form of Shs1 generated in vitro was phosphorylated on eight non-consensus sites in addition to the consensus sites.

**Table 1:** Phosphorylation sites detected in the Shs1 protein

Peptide Sequence	Site(s)	In vitro (partial)	In vitro (full)	In vivo
Ac-STAST*PPINLFR	T6			+
TAST*PPINLFR	T6	+	+	
SNASI(SS)*NPEVK	S63 (S64)			+
ADS*FTKEELTQFR	S221			+
KFEVDPEDDDLES*MEENQAL	S259			+
LSSVANAEEIGPN(ST)*KR	S350 (T351)		+	
RSEKLSSVANAEEIGPN(ST)*KRQSNAPSLSNF	S350 (T351)		+	
SSVANAEEIGPN(ST)*KRQSNAPSLSNF	S350 (T351)	+	+	
SSVANAEEIGPNSTKRQS*NAPSLSNF	S355			+
STKRQS*NAPS*LSNF	S355, S359			+
RQSNAPS*LSNF	S359			+
SSVANAEEIGPNSTKRQSNAPS*LSNF	S359			+
STKRQSNAPS*LSNF	S359			+
KRQSNAPS*LSNF	S359			+
ASLIST*GQFNSSQTL	S369	+		
ASLISTGQFN(SS)*QTL	S374 (S375)			+
ISTGQFN(SS)*QTL	S374 (S375)			+
ANNLRADT*PRNQ	T386	+		
ANNLRADT*PRNQVSGNF	T386	+	+	+
RADT*PRNQVSGNF	T386	+	+	+
ADT*PRNQVSGNFK	T386		+	
KENEYEDNGEHDS*AENEQEMSPVRQL	S408			+
ENEYEDNGEHDS*AENEQEMSPVR	S408			+
NQVSGNFKENEYEDNGEHDS*AENEQEMSPVR	S408			+
KENEYEDNGEHDSAENEQEMS*PVRQL	S416		+	+
ENEYEDNGEHDSAENEQEMS*PVR	S416		+	+
NQVSGNFKENEYEDNGEHDSAENEQEMS*PVR	S416		+	+
KTESS*PKFL	S441			+
FLNS*PDLPER	S447	+	+	+
FLNS*PDLPERT*K	S447, T454		+	
NIS*ETVPYVLR	S460		+	+
NISET*VPYVLR	T462		+	
INQNKLNG(SSSS)*IN	S519 (S520, S521, S522)		+	+
INQNKLNG(SSSS)*INSL	S519 (S520, S521, S522)		+	
INQNKLNG(SSSS)*INSLQQSTR	S519 (S520, S521, S522)		+	
LNG(SSSS)*INSLQQSTR	S519 (S520, S521, S522)		+	+
INQNKLNGS*(SSS)*INSLQQSTR	S519, S522 (S520, S521)			+
LNGS*(SSS)*INSLQQSTR	S519, S522 (S520, S521)			+
LNGSSSSINS*LQQSTR	S525		+	
LNGSS(SS)*INS*LQQSTR	S522 (S521), S525			+
LINQNKLNGS*(SSS)*INS*LQQSTR	S519, S520 (S521, S522), S525			+
INQNKLNG(SSSS)**INS*LQQSTR	2 (S519, S520, S521, S522), S525			+
INQNKLNGSSS*SINS*LQQSTR	S521, S525			+
LNGSSSSINSLQQ(ST)*R	S529 (T530)		+	
KNDT*YTDLASIASGR	T539			+
NDT*YTDLASIASGR	T539			+
SQIKKNDT*YTDL	T539			+
SQIKKND(TYT)*DLASIA	T539 (Y540, T541)			+
KND(TYT)*DLASIASGR	T539 (Y540, T541)		+	
KNDTYTDLAS*IASGR	S545		+	+
NDTYTDLAS*IASGR	S545			+
KNDT*YTDLAS*IASGR	T539, S545			+
NDT*YTDLAS*IASGR	T539, S545			+
KNDTYTDLASIAS*GR	S548			+
NDTYTDLASIAS*GR	S548			+
NDT*YTDLASIAS*GR	T539, S548			+
KNDTYTDLAS*IAS*GR	S545, S548			+
NDTYTDLAS*IAS*GR	S545, S548			+
KNDT*YTDLAS*IAS*GR	T539, S545, S548			+
NDT*YTDLAS*IAS*GR	T539, S545, S548			+

Two independent digestions (trypsin and chymotrypsin) were performed on Shs1 gel bands, which resulted in high sequence coverage and redundant and therefore more reliable phosphorylation site identifications. Phosphorylation sites are denoted in the peptide sequence by an asterisk. When MS/MS spectra did not contain enough fragment ions to unambiguously assign the phosphorylation site within the peptide sequence, all possibly modified amino acids are grouped in parenthesis in column 1 and also the most likely position is indicated in column 2, followed by alternate positions in parenthesis. The column labeled “In vitro (partial)” refers to the partially phosphorylated form of Shs1 generated in vitro. The column labeled “In vitro (full)” refers to the fully phosphorylated form of Shs1 generated in vitro.

The Shs1-3XHA isolated from yeast cells to map in vivo phosphorylation sites was phosphorylated on a total of nineteen sites, including all five CDK consensus sites. Four of the eight non-consensus sites that were phosphorylated by Pho85-Pcl1 in vitro were also phosphorylated in vivo. The in vivo mapping experiments may have missed some sites because they were carried out with total Shs1-3XHA isolated from asynchronous cells, rather than with specific isoforms, to ensure that there was no bias towards specific phosphorylation sites. As a result, the Shs1-3XHA used for in vivo mapping was not quantitatively phosphorylated, whereas the Shs1 phosphorylated in vitro was quantitatively phosphorylated, which may have led to better detection of some phosphorylation sites on the in vitro phosphorylated form of Shs1.

To assess the significance of Pho85-Pcl1 dependent phosphorylation of Shs1, we generated four phosphorylation site mutants of Shs1 (Figure 6A). In one version, we converted the two Pho85 consensus sites (S/TPXI/L) to alanines (referred to as *shs1-ps1*) [40]. In another version, we converted all five minimal CDK consensus sites to alanines (*shs1-ps2*). We also made two mutant versions of Shs1 in which we mutated non-consensus sites in addition to the five consensus sites. In *shs1-ps3*, we mutated only the non-consensus sites that were phosphorylated in vitro and in vivo. In *shs1-ps4,* we mutated all non-consensus sites that were phosphorylated in vitro. In the mapping experiments we sometimes identified phosphorylation sites that occurred at two or more adjacent serines or threonines, and it was impossible to unambiguously identify which serine or threonine was phosphorylated. In these cases, it seemed likely that the adjacent serines or threonines could be phosphorylated in vivo. We therefore mutated each of the adjacent serines or threonines at the site to ensure that all phosphorylation would be eliminated. All four *shs1-ps* mutants were integrated at the endogenous *SHS1* locus.

To test the effects of these phosphorylation site mutants on Shs1 phosphorylation, we assayed Shs1 phosphorylation in log phase populations of wild type, *shs1-ps1*, *shs1-ps2*, *shs1-ps3*, and *shs1-ps4* cells (Figure 6B). All mutants showed a loss of phosphorylation, each one varying depending on the number and type of mutation. The mutant versions of Shs1 in which only the CDK consensus sites were mutated to alanines (*shs1-ps1* and *shs1-ps2*) showed a decrease in the most hyperphosphorylated upper form of Shs1, whereas the mutant versions that also included the non-consensus site mutations (*shs1-ps3* and *shs1-ps4*) showed a more dramatic loss of phosphorylation. These results demonstrate that both CDK consensus and non-consensus sites are phosphorylated in vivo. Note that the *shs1-ps4* mutant was less phosphorylated than the *shs1-ps3* mutant. This suggests that the four non-consensus sites that were mapped in vitro, but not in vivo, are genuine phosphorylation sites in vivo. We also tested whether the upper form of Shs1 that remained in *shs1-ps1* and *shs1-ps2* became hyperphosphorylated during the cell cycle (Figure 6C). The levels of the upper form changed in both *shs1-ps1* and *shs1-ps2* during the cell cycle, but in the *shs1-ps1* mutant, which retains three functional consensus sites, the upper form became hyperphosphorylated at 40 minutes, similar to wild type.

Mutation of the sites that were phosphorylated by Pho85-Pcl1 in vitro caused a much greater loss of in vivo phosphorylation than *pho85Δ*. Moreover, the loss of phosphorylation observed for the *shs1-ps3* mutant closely resembled the loss of phosphorylation observed in *cln1Δ cln2Δ GAL1-CLN3* cells after repression of *CLN3* transcription. Since Cln3 plays a role in pathways that initiate transcription of Pcl1, Pcl2, Cln1, and Cln2, these observations suggest that Cdk1 and Pho85 associated with G1 cyclins act redundantly to phosphorylate Shs1 in vivo [3], [35], [36].

### Known functions of Shs1 are dependent upon phosphorylation

We next characterized the functional significance of Shs1 phosphorylation. Surprisingly, we found that the *shs1-ps* mutants caused little or no phenotype in *cln1Δ GAL-CLN2* cells when grown in dextrose. In addition, the *shs1-ps* mutants localized to the bud neck (Figure S1). These observations demonstrate that CDK-dependent phosphorylation of Shs1 is not required for the roles that Shs1 plays in regulating the pattern of cell growth or septin localization early in the cell cycle. These observations also show that the *shs1-ps* mutants retain key functions, which suggests that the mutations did not cause non-specific loss of function.

**Figure 7:**
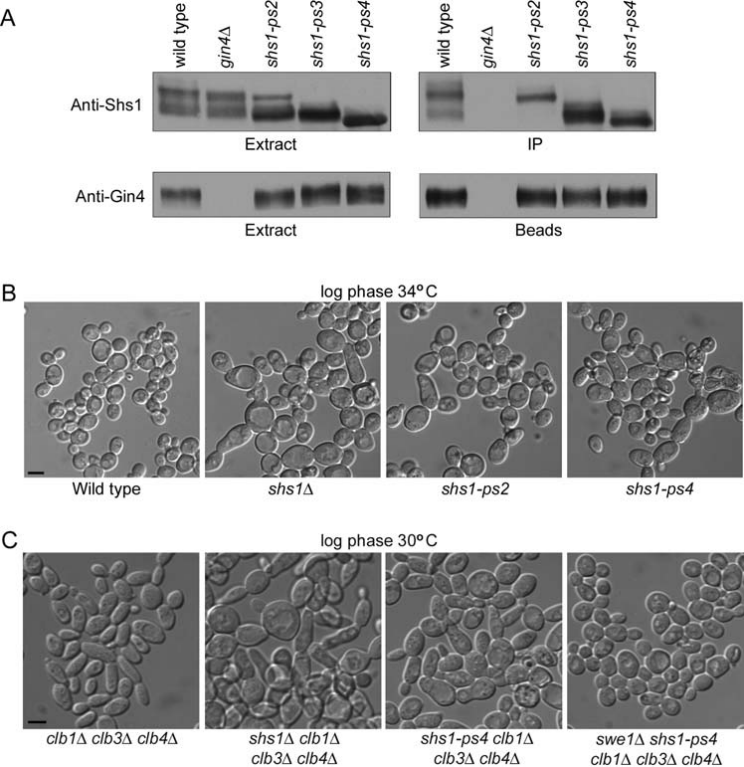
Phosphorylation of Shs1 regulates association of Shs1 with the Gin4 kinase and causes increased cell size and defects in cell morphology that are due to delayed entry into mitosis. (A) Extracts made from wild type, *gin4Δ*, *shs1-ps2*, *shs1-ps3*, and *shs1-ps4* cells were arrested in mitosis with benomyl, and Gin4 was immunoprecipitated using an affinity purified anti-Gin4 polyclonal antibody. Co-precipitation of and Shs1 was assayed by Western blotting. The amount of Gin4 in the extracts and bound to the beads was assayed by Western blotting. (B) Cells of the indicated genotypes were grown in YPD media to log phase at 34°C and photographed. Bar, 5 µm for all panels. (C) *clb1Δ clb3Δ clb4Δ*, *shs1Δ clb1Δ clb3Δ clb4Δ*, *shs1-ps4 clb1Δ clb3Δ clb4Δ*, and *swe1Δ shs1-ps4 clb1Δ clb3Δ clb4Δ* cells were grown to log phase in YPD media at 30°C and photographed. Bar, 5 µm for all panels.

We next tested whether the *shs1-ps* mutants have effects on the interaction of Shs1 with the Gin4 kinase, since previous work found that Shs1 and the other septins associate with Gin4 in an Shs1-dependent manner when cells enter mitosis [13]. We immunoprecipitated Gin4 from wild type, *shs1-ps2, shs1-ps3, and shs1-ps4* cells that were arrested in mitosis and probed for the presence of associated Shs1 (Figure 7A). The *shs1-ps2* protein was found predominantly in a hypophosphorylated lower form in cells arrested in mitosis, and this form failed to associate with Gin4. In contrast, a hyperphosphorylated upper form of *shs1-ps2* bound efficiently to Gin4. The *shs1-ps3* protein associated with Gin4 with high efficiency, while *shs1-ps4* showed a reduced association with Gin4 when compared to *shs1-ps3*. These results suggest that Shs1 phosphorylation plays a role in regulating the association of Shs1 with Gin4. However, the mechanisms by which Shs1 phosphorylation controls association with Gin4 appear to be complex. One model that could explain these observations is that phosphorylation of a subset of sites promotes association of Gin4 with Shs1, while phosphorylation of other sites inhibits the association.

Loss of the Gin4 kinase causes a prolonged G2/M delay [13], [17], [41], [42]. Polar growth continues during the delay, which leads to growth of cells that are larger and more elongated than wild type cells. The delay is caused by a failure to inactivate the Swe1 kinase, which blocks entry into mitosis by phosphorylating and inhibiting Cdk1 [41]. Thus, the G2/M delay and cell size defects caused by *gin4Δ* are rescued by *swe1Δ*. Conversely, the phenotype of *gin4Δ* is strongly enhanced in *clb1Δ clb3Δ clb4Δ* cells, which have reduced mitotic Cdk1 activity because they lack redundant mitotic cyclins [42]. Previous work found that *shs1Δ* causes an increased cell size and cell elongation phenotype that is similar to the phenotype caused by *gin4Δ* [17], [18]. Moreover, the phenotype of *shs1Δ* is enhanced in *clb1Δ clb3Δ clb4Δ* cells [17]. We therefore tested whether the *shs1-ps* mutants cause a phenotype that is similar to *shs1Δ*. We found that the *shs1-ps* mutants were significantly larger than wild type cells and slightly elongated at 34°C, although the elongated cell phenotype was not as penetrant as the phenotype caused by *shs1Δ* (Figure 7B). In addition, the phenotype of *shs1-ps4* was enhanced in cells that are dependent upon the Clb2 mitotic cyclin for survival, as observed for *shs1Δ* (Figure 7C). The phenotype of *shs1-ps4 clb1Δ clb3Δ clb4Δ* cells was rescued by *swe1Δ*, which indicates that *shs1-ps4* causes a Swe1-dependent G2/M delay. Thus, failure to phosphorylate Shs1 causes a failure to inactivate Swe1 in a timely manner at the G2/M transition.

## Discussion

### The septins function in redundant pathways that control the pattern of cell growth early in the cell cycle

A number of observations suggest that the septins function in pathways initiated by G1 cyclins to control the pattern of cell growth. The septins show strong synthetic lethal interactions with redundant G1 cyclins, and loss of Shs1 or Cdc12 in *cln1Δ cln2Δ* cells causes defects in formation of a focused bud neck and a failure to restrict growth to the daughter cell. Since the bud neck is formed in late G1, it seems likely that the septins execute functions when they first localize to the site of bud emergence in G1. The finding that Shs1 is required for normal localization of Cdc11 in unbudded cells that lack Cln1 and Cln2 further supports the idea that the septins carry out functions in G1. Finally, previous work found that G1 CDK activity plays a role in localizing the septins to a ring early in the cell cycle, which demonstrates that the septins are targets of pathways initiated by G1 cyclins [43], [44]. These observations might be explained by the existence of redundant septin-dependent and septin-independent pathways that control formation of the bud neck and the pattern of bud growth. The genetic interactions between the septins and *cln1Δ cln2Δ* suggest that the septin-independent pathway is initiated primarily by Cln1 and Cln2, while the septin-dependent pathway is initiated by Pcl1 and Pcl2. Recent studies found that the *C. albicans* homolog of Cln1 targets Cdk1 activity to the Cdc11 septin, which further suggests the existence of CDK-dependent pathways that regulate septin function during G1 [45].

Interestingly, *shs1Δ* is also synthetically lethal with a member of the formin family of proteins [46]. There are two formins in budding yeast that are referred to as Bni1 and Bnr1 [47]–[51]. Bni1 mediates nucleation of actin filaments within the bud that guide vesicles to sites of polarized growth, while Bnr1 mediates nucleation of actin filaments at the bud neck that may play a role in guiding vesicles to the bud neck [52]. Loss of Bni1 causes a widened bud neck and reduced polar cell growth, while loss of Bnr1 causes only a slight delay in cell separation [49], [53]–[57]. Loss of both Bni1 and Bnr1 is lethal and causes severe defects in bud formation, which indicates that they carry out overlapping functions in events required for bud formation [47], [48], [58]. Bnr1-dependent nucleation of actin filaments is dependent upon the septins [52]. Moreover, *bni1Δ* is synthetically lethal with *shs1Δ* and with *pho85Δ* [46], [59]. Thus, Shs1 may be required for Bnr1-dependent nucleation of actin filaments, which would explain the synthetic lethal interaction between *shs1Δ* and *bni1Δ*. Taken together, these genetic interactions suggest that Shs1 and the other septins may play a role in redundant formin-dependent pathways that help direct secretory vesicles to the correct locations during cell growth. Experiments in vertebrate cells have also suggested that the septins carry out roles in the secretory pathway [26], [28]–[30]. Alternatively, the septins might function as a scaffold for recruitment of proteins that are required for Bnr1-dependent nucleation of actin filaments, or as a diffusion barrier that restricts localization of proteins required for formation of a new bud to the site of bud emergence.

### Shs1 is phosphorylated by Pho85-Pcl1 on consensus and non-consensus sites

Pho85 phosphorylates Shs1 on non-consensus sites in vitro and the same sites are phosphorylated in vivo. Since Cdk1 acts redundantly with Pho85 to phosphorylate Shs1, it is likely that Cdk1 phosphorylates the same non-consensus sites. Previous work provides another example of phosphorylation of non-consensus sites by a CDK [60]. In this case, Cdk1-Clb2 was found to phosphorylate both consensus sites and non-consensus sites on the Swe1 kinase. Phosphorylation of the non-consensus sites was detected both in vivo and in vitro, and phosphorylation of non-consensus sites was dependent upon phosphorylation of consensus sites in vivo. Phosphorylation of consensus sites on Swe1 therefore appears to facilitate phosphorylation of non-consensus sites. More recent experiments have similarly found that Cdk1-Cln2 phosphorylates Boi1 on both consensus and non-consensus sites, and that phosphorylation of consensus sites is required for phosphorylation of non-consensus sites in vivo [6]. These observations could be explained by a model in which phosphorylation of consensus sites creates a binding site for Cdk1-cyclin, which generates a high local concentration of Cdk1-cyclin that drives phosphorylation of non-consensus sites that would otherwise be kinetically unfavored. The Clb2 cyclin has been shown to bind to phosphorylated Cdk1 consensus sites, which supports such a model [61].

A number of observations suggest that phosphorylation of Shs1 on consensus sites may work similarly to facilitate further phosphorylation of non-consensus sites. In the in vitro phosphorylation experiments we recovered a form of Shs1 that was phosphorylated primarily on consensus sites, and a second form that was phosphorylated on both consensus sites and non-consensus sites, which suggests that phosphorylation of consensus sites is required for phosphorylation of non-consensus sites. We also found that mutation of consensus sites caused a significant reduction in the amount of fully hyperphosphorylated Shs1 in vivo. Since the fully hyperphosphorylated form of Shs1 corresponds to the form that is phosphorylated on non-consensus sites, this suggests that failure to phosphorylate consensus sites causes a significant reduction in phosphorylation of non-consensus sites.

Some phosphorylated forms of Shs1 that are dependent upon CDK activity are present throughout the cell cycle, but Pcl1, Pcl2, Cln1, and Cln2 are only present during G1. Phosphorylation of Shs1 at times other than G1 may be due to Cdk1-Cln3, which is thought to be active throughout the cell cycle [35]. Interpretation of the role of Cln3 in Shs1 phosphorylation is complicated by the fact that Cln3 functions in a pathway that helps trigger transcription of the other G1 cyclins [35], [36]. Thus, Cdk1-Cln3 could play important roles in Shs1 phosphorylation by directly phosphorylating Shs1 and by helping initiate transcription of the other G1 cyclins.

All phosphorylation of Shs1 by Pho85-Pcl1 occurs outside of the known functional domains of Shs1. It therefore seems unlikely that phosphorylation of Shs1 by Pho85-Pcl1 directly regulates the intrinsic GTP or phosphoinositide binding activities of Shs1. Comparison of the in vitro and in vivo phosphorylation site mapping experiments revealed that kinases other than Pho85 phosphorylate the GTP-binding domain of Shs1. The Gin4 and Rad53 kinases have been found to phosphorylate Shs1 and may be responsible for phosphorylation of these sites [13], [62].

### Phosphorylation of Shs1 by G1 cyclin-CDKs is required for regulation of events that occur during G2/M

Since *shs1Δ* is synthetically lethal with *cln1Δ cln2Δ* and is phosphorylated by Pho85-Pcl1, we initially hypothesized that Shs1 is downstream of Pho85-Pcl1 in a pathway that helps control bud neck formation and the pattern of growth. Surprisingly, however, we found that *shs1-ps* mutants were not synthetically lethal with *cln1Δ cln2Δ* and had no effect on the localization of Cdc11. Rather, the *shs1-ps* mutants caused an increased cell size and a failure to suppress polar growth that appeared to be due to a Swe1-dependent G2/M delay.

The *shs1-ps* mutants also caused complex effects on the association of Shs1 with the Gin4 kinase during mitosis. Previous work found that loss of Gin4 causes a Swe1-dependent G2/M delay that leads to formation of elongated cells [42], [63]. Moreover, the phenotype of *gin4Δ* is strongly enhanced in Clb2-dependent cells [42]. The similar phenotypes of *gin4Δ* and the *shs1-ps* mutants, combined with the finding that phosphorylation of Shs1 appears to regulate association with Gin4, suggests that interactions between Gin4 and Shs1 may be required for normal progression through G2/M.

One might imagine several models that could explain the role of phosphorylation of Shs1 by G1 cyclin-CDKs. Phosphorylation of Shs1 could regulate recruitment of Gin4 or other proteins that function to redirect growth away from the bud tip, thereby ending polar growth. Other possible models involve checkpoint functions for phosphorylation of Shs1. For example, previous work suggested the existence of a checkpoint that monitors septin assembly and induces a Swe1-dependent G2/M delay in response to defects in septin assembly [63], [64]. Shs1 phosphorylation could therefore generate a positive signal that indicates when septin assembly has occurred normally. However, a complete loss of septin function caused by temperature sensitive alleles of Cdc3 or Cdc12 causes a prolonged G2/M delay, whereas *gin4Δ*, *shs1Δ,* or *shs1-ps* cause only a mild G2/M delay [11], [17], [42]. Therefore, phosphorylation of Shs1 cannot be the only signal that indicates that septin assembly is normal.

Phosphorylation of Shs1 by G1 cyclin-CDKs could also be part of a checkpoint that monitors polar bud growth. Polar growth is initiated by G1 cyclin-CDKs and must be terminated when sufficient growth has occurred. Phosphorylation of Shs1 by G1 cyclin-CDKs could help link successful completion of polar growth events to entry into mitosis. In this case, polar growth would be expected to continue inappropriately in *shs1-ps* mutants, which would lead to growth of cells that are slightly elongated and abnormally large. These kinds of checkpoint mechanisms must exist to coordinate cell growth with the cell cycle, yet little is known about them. The fact that the septins are required for localization of Bnr1 and for the normal pattern of growth in *cln1Δ cln2Δ* cells demonstrates that the septins are involved in growth-related events and may therefore be good candidates for proteins that help monitor growth. Further analysis of the function and regulation of Shs1 phosphorylation may therefore provide clues to how cells coordinate cell growth with the cell cycle.

## Materials and Methods

### Media and strains

Standard yeast media were used. YPD and YPGal media were supplemented with 40 mg/liter of adenine. All strains are isogenic to W303 (*leu2-3,112 ura3-52 can1-100 ade2-1 his3-11 trp1-11 ssd1*). The additional features of the strains used in this study are listed in Table 2. DK516 and DK1032 were generated using standard genetic crosses and tetrad analysis. To create DK515 and DK548, the *GAL1* promoter and 3XHA was integrated upstream of *CLN2* using standard procedures (oligos: ACTCTATAGCTGCCAATTCATTCGCTTAC-CACATCATAATGAATTCGAGCTCGTTTAAAC and TGATGACGAGTCCCAT-ACGGGGTCTTGGTTCAGCACTAGCGCACTGAGCAGCGTAATCTG) [65]. To create DK1080, DK1096, and DK1106, three repeats of the HA epitope were integrated downstream of *SHS1* using standard procedures (oligos: TTATTTATTTGCTCAGCTTTGGATTTTGTACAGATACAACGAATTCGAGCTCGTTTAAAC with CACGTATACTGATTTAGCCTCTATTGCATCGGGTAGAGATCGGAT-CCCCGGGTTAATTAA for DK1080, or with CGCGTATGCTGATTTAGCCGCTA-TTGCATCGGGTAGAGATCGGATCCCCGGGTTAATTAA for DK1096 or with CACGTATACTGATTTAGCCGCTATTGCATCGGGTAGAGATCGGATCCCCGG-GTTAATTAA for DK1106). DK573 was made by using PCR to amplify *pcl1Δ::natMX6* from BY1404 (gift from Brenda Andrews) and *pcl2Δ::kanMX6* from the yeast haploid deletion collection (Open Biosystems) followed by transformation of the PCR products into DK186 using standard procedures. Similarly, DK1051 was made by using PCR to amplify *swe1Δ::URA* from SH24 followed by transformation of the PCR product into DK1031. To create DK1068, a plasmid (pCLN2-HA) that contained Cln2 fused to three repeats of the HA epitope was linearized with *Pvu*II to target integration at the *CLN2* gene and then transformed into DK573. To create the *shs1-ps* mutants a plasmid that included the wild type *SHS1* gene was made by using PCR to amplify the *SHS1* open reading frame and flanking control regions (oligos: GCGGGATCCGCGACTTGA-ACCATTCAGTC and GCGGCATGCGAAGTTACGGGAAATCATGATAG). This fragment was then cloned into the *Bam*H1 and *Sph*1 sites of the YCPlac111 vector to create pTE2. pTE2 was mutagenized by site-directed mutagenesis to convert T6 to an alanine to create pTE15. To create *shs1-ps1*, pTE15 was further mutagenized to convert S447 to an alanine (pTE17). To create *shs1-ps2*, pTE15 was cut with restriction enzymes *Msc*1 and *Bsp*EII to drop out a 328 bp fragment. This fragment was replaced with a synthesized fragment that contained mutations of the four consensus sites at T386, S416, S441, and S447 to alanines (pTE13). The *shs1-ps3* and *shs1-ps4* alleles were constructed by using PCR to amplify a 1308 bp fragment (oligos: CCGCGATAAAATTGCTCAA-TTGGCACCA and ATTAGGACCAATTTCTTCAGCGTTGGCC) of pTE15 and a 787 bp fragment (oligos: GGCCAACG-CTGAAGAAATTGGTCCTAAT and TATGTGTGTTCCCTTCTTGAAGGCTGTG) from plasmids pTE18 and pTE19. pTE18 contained a synthesized fragment that included mutations of the twelve sites at T386, S416, S441, S447, S460, T462, S519, S520, S521, S522, S525, and S545 to alanines and was used to make *shs1-ps3*. pTE19 contained a synthesized fragment that included mutations of the nineteen sites at S350, T351, T386, S416, S441, S447, T454, S460, T462, S519, S520, S521, S522, S525, S529, T530, T539, T541, and S545 to alanines and was used to make *shs1-ps4*. These PCR fragments were gel purified and pieced together by PCR amplification (oligos: CCGCGATAAAATTGCTCAATTG-GCACCA and TATGTG-TGTTCCCTTCTTGAAGGCTGTG) to create full length *shs1-ps3* and *shs1-ps4*. The *shs1-ps* mutants were integrated into the genome at the *SHS1* locus as previously described [60]. The entire open reading frame of all *shs1-ps* mutants was verified by sequencing. All gene synthesis was carried out by DNA 2.0, Menlo Park, California.

**Table 2:** Strains used in this study

Strain	Genotype	Reference or Source
CC7	*MATα, bar1Δ, cdc12-6*	This study
CC10	*MATa, bar1Δ, SHS1-3XHA-URA3*	[17]
DK186	*MATa, bar1Δ*	[42]
DK212	*MATa, bar1Δ, clb1Δ, clb3Δ::TRP1, clb4Δ::HIS3*	[42]
DK223	*MATa, bar1Δ, clb1Δ, clb3Δ::TRP1, clb4Δ::HIS3 clb2Δ::LEU2 ura3::GAL10-CLB2*	This study
DK252	*MATa, bar1Δ, cln1Δ::his5+, cln2Δ::LEU2, ura3::GAL1-CLN3*	Gift from Bruce Futcher
DK515	*MATa, bar1Δ, shs1Δ::URA3, cln1Δ::TRP1, GAL1-CLN2-3XHA his5+*	This study
DK516	*MATa, bar1Δ, cdc12-6, cln1Δ::TRP1, cln2Δ::LEU2*	This study
DK548	*MATa, bar1Δ, cln1Δ::TRP1, GAL1-CLN2-3XHA his5+*	This study
DK573	*MATa, bar1Δ, pcl1Δ::natMX6, pcl2Δ::kanMX6*	This study
DK747	*MATα, bar1Δ, shs1Δ::URA3*	This study
DK912	*MATa, bar1Δ, shs1::shs1-ps2*	This study
DK966	*MATa, bar1Δ, shs1::shs1-ps1*	This study
DK985	*MATa, bar1Δ, shs1::shs1-ps4*	This study
DK1031	*MATα, bar1Δ, shs1::shs1-ps4*	This study
DK1032	*MATa, bar1Δ, shs1::shs1-ps4, clb1Δ, clb3Δ::TRP1, clb4Δ::HIS3*	This study
DK1033	*MATa, bar1Δ, shs1::shs1-ps3*	This study
DK1051	*MATa, bar1Δ, shs1::shs1-ps4, clb1Δ, clb3Δ::TRP1, clb4Δ::HIS3, swe1Δ::URA3*	This study
DK1068	*MATa, bar1Δ, pcl1Δ::natMX6, pcl2Δ::kanMX6, CLN2-3XHA-LEU2*	This study
DK1080	*MATa, bar1Δ, shs1::shs1-ps2-3XHA his5+*	This study
DK1096	*MATa, bar1Δ, shs1::shs1-ps4-3XHA kanMX6*	This study
DK1106	*MATa, bar1Δ, shs1::shs1-ps3-3XHA his5+*	This study
KA61	*MATa, bar1Δ, cln1Δ::TRP1, cln2Δ::LEU2*	This study
RA19	*MATa, bar1Δ, gin4Δ::LEU2*	This study
RA25	*MATa, bar1Δ, shs1Δ::URA3, clb1Δ, clb3Δ::TRP1, clb4Δ::HIS3*	[17]
SH24	*MATa, bar1Δ, swe1Δ::URA3*	[71]
SH183	*MATa, bar1Δ, pho85Δ::kanMX6*	This study
SH184	*MATa, bar1Δ, cln3Δ::HIS5*	This study
ZZ41	*MATa, bar1Δ, CLN2-3XHA-LEU2*	[72]

### Immunofluorescence, cell cycle arrests, and centrifugal elutriation

Fixation and staining of cells with antibodies was carried out as previously described [66]. Cells were arrested in G1 by addition of 0.5 µg/ml α factor to log phase cultures, followed by growth at room temperature for 3 hours. Small, unbudded cells were isolated from log phase cells by centrifugal elutriation in a Beckman J6-MI centrifuge and a JE-5.0 rotor as previously described [67].

### Sample preparation, PAGE, and Western blotting

Samples for time courses were collected as previously described with the exception that glass beads were added before freezing and 115 µl of 1X sample buffer supplemented with 2 mM PMSF, 50 mM NaF, 50 mM β-glycero-phosphate, and 5% β-mercaptoethanol was added to frozen pellets before bead beating [42]. The phosphorylated forms of Shs1 were resolved on a 10% polyacrylamide gel (14 cm wide×9 cm long×1 mm thick) for 3.5 hours at constant current (20 mA) [68]. Western blot transfers were carried out for 2 hours at 400 mA at 4°C in a Hoeffer transfer tank in a buffer containing 200 mM tris base, 1.5 M glycine, and 20% methanol.

### Immunoaffinity purifications of Shs1-3XHA

Immunoaffinity purification of Shs1-3XHA for in vitro phosphorylation by Pho85-Pcl1 was carried out in the presence of 1 M KCl using the same protocol used previously to purify Gin4, with the following changes [13]. To purify Shs1-3XHA without lambda phosphatase treatment, cells were lysed in a buffer that contained 50 mM HEPES-KOH, pH 7.6, 1 M KCl, 75 mM β-glycero-phosphate, 75 mM NaF, 1 mM MgCl_2_, 1 mM EGTA, 0.45% Tween-20, 10% glycerol, 1 mM phenylmethylsulfonyl fluoride (PMSF) and the column was washed in the same buffer without PMSF. The elution buffer was the same as the lysis buffer except that it contained 0.5 mg/ml HA dipeptide, 0.01% Tween-20, and no PMSF. To purify Shs1-3XHA treated with lambda phosphatase, cells were lysed in buffer containing 50 mM HEPES-KOH, pH 7.6, 1 M KCl, 1 mM MgCl_2_, 1 mM EGTA, 0.45% Tween-20, 5% glycerol, 1 mM PMSF. After the extracts were incubated with the immunoaffinity beads, the beads were washed twice batchwise with 15 ml of lysis buffer and then twice with 15 ml of phosphatase buffer (50 mM Tris-HCl, pH 7.5, 5 mM DTT, 2 mM MnCl_2_, 100 µg/ml BSA). The beads were transferred to a 1.6 ml tube and then washed 5 times with 1 ml of phosphatase buffer. The beads were pelleted, the supernatant was removed, and the beads were incubated with 20 µL of lambda phosphatase (New England Biolabs) for 45 min at 30°C, with gentle mixing every 5 min. The beads were transferred to a 1.5-ml Biospin column (Bio-Rad) and washed with 5 ml of wash buffer (50 mM HEPES-KOH, pH 7.6, 200 mM KCl, 1 mM MgCl_2_, 1 mM EGTA, 5% glycerol, 0.01% Tween-20) by pipeting 1-ml aliquots of buffer on top of the column and allowing the buffer to flow through by gravity. The column was eluted as described for the Shs1-3XHA that was not treated with phosphatase.

### Purification of 6XHIS-Pho85/GST-Pcl1 complexes and 3XHA-Cln2/Cdk1

Full length *PCL1* was amplified by PCR and cloned into a Gateway entry vector (pDONR221) to create pTE4 (oligos for first round of PCR: AACCTGTACTTCCAGTCCATGTGTGAATACAGCAAGGC and GTACAAGAAA-GCTGGGTCCTAAAACCCATGTTGACTCATG and oligos for second round of PCR: GGGGACAAGTTTGTACAAAAAAGCAGGCTCCGAAAACCTGTACTTCC-AG and GGGGACCACTTTGTACAAGAAAGCTGGGTCGGACTGGAAGTACAG-GTT). The DNA was verified by sequencing and then recombined into a Gateway destination vector (pDEST15) to fuse glutathione-S-transferase (GST) to the N-terminus of Pcl1 (pTE6). Full length *PHO85* from a cDNA library was amplified by PCR and cloned into Gateway entry vector PCR8/TOPOA to create pTE7 (oligos: CGCATGTCTTCTTCTTCACAATTTAAGC and CGCTTATGAAGCGT-GGTGGTAGTAC). The DNA was verified by sequencing and then recombined into a Gateway destination vector (pDEST17 with AMP replaced with KAN, gift from John Little) to fuse six histidines at the N-terminus of Pho85 (pTE9). pTE6 and pTE9 were co-transformed into BL21 Arabinose cells and plated on LB plates containing 50 µg/ml kanamicin and 100 µg/ml ampicillin. Cells carrying these plasmids were grown to an optical density of 1.0 at 37°C, chilled on ice until they reached 25°C, and then induced with 0.2% L-Arabinose for 4 hours at room temperature. Cells were pelleted, frozen in liquid nitrogen, and stored at −80°C. Purification of the 6XHIS-Pho85/GST-Pcl1 complex was carried out by successive glutathione and nickel affinity chromatography as previously described for Gin4, with the following changes [17]. The glutathione affinity chromatography was performed as previously described, with the exception that the column was eluted with a buffer containing 50 mM phosphate, pH 8.0, 100 mM NaCl, 5 mM reduced glutathione [69]. The eluted 6XHIS-Pho85/GST-Pcl1 complex was loaded directly onto a 1 ml Ni-NTA column, washed with 20 column volumes 50 mM phosphate pH 8.0, 500 mM NaCl, 5 mM β-mercaptoethanol, 10 mM imidizole, and eluted with 50 mM phosphate pH 7.0, 500 mM NaCl, 250 mM imidizole pH 7.0. The yield from 4 liters of culture was ∼1.5 mg. Peak fractions were pooled and dialyzed in 75 mM HEPES-KOH, pH 7.6, 10% glycerol, 1 mM DTT, 0.05% Tween-20, 1 mM MgCl_2_ and frozen in liquid nitrogen. 3XHA-Cln2/Cdk1 was purified by immunoaffinity chromatography and used in kinase assays as previously described [70].

### Kinase assays

To demonstrate that Shs1 is directly phosphorylated by Pho85-Pcl1, increasing amounts of purified 6XHIS-Pho85/GST-Pcl1 were added to the purified dephosphorylated Shs1-3XHA in the presence of kinase assay buffer (50 mM HEPES-KOH, pH 7.6, 2 mM MgCl2, 0.05% Tween-20, 10% glycerol, 1 mM DTT, 1 mM ATP). The reactions were incubated at 30°C for 1 hour and terminated by the addition of 2.5 µl of 4X sample buffer. The samples were incubated at 100°C for 5 minutes and loaded onto a 10% SDS polyacrylamide gel, which was transferred to nitrocellulose and probed with α-HA antibody. Reactions with purified 3XHA-Cln2/Cdk1 were carried out in a similar manner.

### Phosphorylation-site mapping by Tandem Mass Spectrometry

To map in vitro phosphorylation sites on Shs1, we scaled up the reactions in Figure 5C, lane 4. Both hyperphosphorylated Shs1 protein bands and an unphosphorylated control band were excised from the gel. Phosphorylation sites were mapped on all three forms of Shs1. No phosphorylation sites were identified on the unphosphorylated control sample. To map in vivo sites on Shs1, Shs1-3XHA was affinity purified in the presence of phosphatase inhibitors to prevent dephosphorylation of Shs1, as previously described for purification of in vivo phosphorylated Swe1 [60].

Coomassie blue-stained bands corresponding to Shs1 protein were reduced, carboxiamidomethylated, and digested independently with trypsin and chymotrypsin for increased sequence coverage. Peptide mixtures were separated by microcapillary (125 µM×18 cm) reverse-phase (MagicC18AQ) chromatography and online analyzed on a hybrid mass spectrometer (LTQ-Orbitrap or LTQ-FT, Thermo Electron), in a data-dependent fashion. Precursor masses were collected at high resolution; MS/MS spectra were triggered for the ten most abundant ions and acquired in the linear ion trap. MS/MS spectra were searched using the Sequest algorithm with serine, threonine, and tyrosine phosphorylation and methionine oxidation as dynamic modifications. Peptide matches obtained were deemed correct after applying several filtering criteria, including mass error <10 ppm, tryptic ends for trypsin digested samples. Redundant identifications of phosphorylation sites derived from different digestions added confidence to our results. Further, all spectra corresponding to phosphopeptides were manually inspected for correct sequence identification validation and site assignment based on the presence of site determining fragment ions. Ambiguity is denoted when applicable (see Table 1 legend).

### Coimmunoprecipitation of Gin4 and Shs1

Immunoaffinity beads for the precipitation of Gin4 were made as previously described [13]. To prepare cells for immunoprecipitation experiments, 50 ml of cells at OD_600_ 0.7 were resuspended in YPD containing 30 µg/ml benomyl, followed by growth at room temperature for 3 hours. Cells were pelleted, resuspended in 3 ml of 50 mM HEPES-KOH, pH 7.6, and aliquoted into two 1.6-ml screw-top tubes, pelleted again, and frozen in liquid nitrogen after removing the supernatant. Extracts and immunoprecipitation were carried out as previously described [13].

## Supporting Information

Figure S1 Shs1 phosphorylation site mutants localize normally. shs1-ps2-3XHA, shs1-ps3-3XHA, and shs1-ps4-3XHA cells were grown to log phase. Shs1 localization was determined with an anti-HA antibody.
(4.56 MB TIF)
