# Supplementary figures and images for: The Septins Function in G1 Pathways that Influence the Pattern of Cell Growth in Budding Yeast
Source: PLoS One. 2008 Apr 23;3(4):e2022. doi: 10.1371/journal.pone.0002022 (PMC2291192; doi:10.1371/journal.pone.0002022)

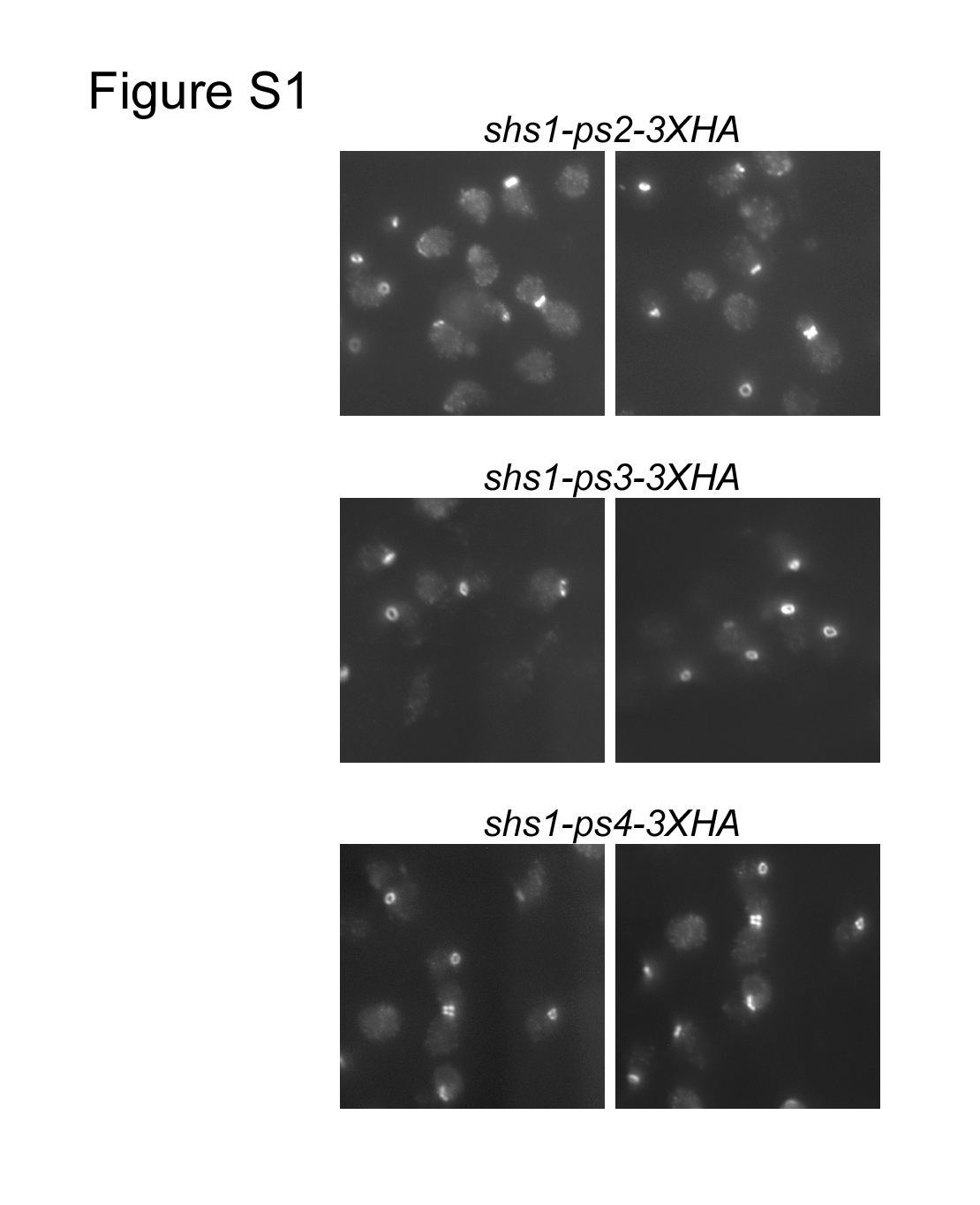

Supplement: Figure S1 — Shs1 phosphorylation site mutants localize normally. shs1-ps2-3XHA, shs1-ps3-3XHA, and shs1-ps4-3XHA cells were grown to log phase. Shs1 localization was determined with an anti-HA antibody. (4.56 MB TIF) [file pone.0002022.s001.tif]
